# Raw, centile and individualised minimal clinically important differences for the MMN-Rasch-built Overall Disability Scale^©^ in the monitoring of multifocal motor neuropathy

**DOI:** 10.1007/s00415-026-13771-8

**Published:** 2026-04-26

**Authors:** Yusuf A. Rajabally, Ahmad Al-Areed, Roshan Iqbal, Muhammed A. Noushad, Young G. Min, Chinar Osman

**Affiliations:** 1 Aston Medical School, Aston University, Birmingham, UK; 2 Inflammatory Neuropathy Service, Department of Neurology, Queen Elizabeth Hospital Birmingham, University Hospitals Birmingham, Birmingham, B15 2TH UK; 3 Wessex Neurological Centre, University Hospital Southampton, Southampton, UK; 4 Department of Neurology, Severance Hospital, Yonsei University College of Medicine, Seoul, Republic of Korea

**Keywords:** Centile, Individualised, Minimal clinically important difference, MMN-RODS, Multifocal motor neuropathy, Outcomes, Raw

## Abstract

**Background:**

Optimal clinical application of the minimal clinically important difference (MCID) for the MMN-Rasch-built Overall Disability Scale^©^ (MMN-RODS) scale is unknown.

**Methods:**

We retrospectively studied subjects with MMN from 2 UK neuropathy centres. Raw and centile MMN-RODS scores were collected, at initial, two intermediate, and latest assessment, and distribution-based MCIDs determined at studied time-points. The sensitivities of raw, centile and individualised MCIDs, were compared.

**Results:**

We included 32 consecutive subjects with MMN on individualised immunoglobulin dosing regimens. First intermediate, second intermediate, and latest assessments were performed at a median of 17.0, 54.1 and 74.6 months from onset, respectively. Progressive amelioration of mean raw MMN-RODS score occurred between initial and latest assessment. The distribution-based raw MMN-RODS MCID was of 3, 4 and 5, and the distribution-based centile MMN-RODS MCID was of 6, 7 and 7, at first intermediate, second intermediate and latest assessment, respectively. At first intermediate assessment, raw and individualised MCIDs were equally sensitive, and both more sensitive than centile MCID (McNemar’s Test: *p* < 0.001, *p* < 0.001). Raw MCID, centile MCID and individualised MCID, all had equivalent sensitivity at the second intermediate and latest assessment. Neither initial MMN-RODS score, nor amplitude of treatment-induced changes in early disease stages, independently predicted total improvement amplitude or final outcome.

**Conclusions:**

MMN-RODS MCID cut-offs may require adaption to assessment timing. In earlier stages, raw/individualised MCID may be more sensitive than centile MCID, whereas all three methods may subsequently be equivalent. Initial disability and early treatment response do not predict achievable total improvement amplitude nor final outcome.

## Introduction

Multifocal motor neuropathy (MMN) is a treatable auto-immune motor neuropathy causing principally upper limb disability [1, 2].

Evaluation of treatment effects in MMN has, from the initial therapeutic studies conducted, relied on strength evaluation through MRC scores. Demonstration of the inadequacy of MRC scores has however convincingly been made, over a decade ago [3], although the scale still remains in use in its previous form. Grip strength has also been utilised, with debatable direct functional significance as well as uncertainty on appropriate minimal clinically important difference (MCID) evaluation methods [4]. The concept of MCID has otherwise itself been gaining importance in the field of inflammatory neuropathy, particularly in chronic inflammatory demyelinating polyneuropathy (CIDP) [5], highlighting the increasing awareness of the significance of clinically meaningful functional benefit, of particular relevance with the use of high-cost therapies such as immunoglobulins, or other newer upcoming treatments.

The MMN-Rasch-built Overall Disability Scale^©^ (MMN-RODS) is a disease specific patient reported outcome measure (PROM) for MMN, published in 2015 [6]. The MMN-RODS evaluates upper limb function through 25 questions, requiring standardised answers (“easy to perform”, “difficult to perform”, “impossible to perform”), producing a raw score ranging from 50 (no disability) to 0 (maximal disability resulting in impossibility to perform all tasks) [6]. This raw score may then be transformed into a centile metric. MCID application for the MMN-RODS scale has not been studied extensively. Individualised MCIDs have been derived and applied in a small cohort, but distribution-based methods have only been used, to our knowledge with centile scores, in one recent study from our units which analysed long-term outcomes and immunoglobulin dosage [7].

We conducted a retrospective study of subjects with MMN treated with individualised immunoglobulin regimens, attending our 2 UK peripheral neuropathy centres, in Birmingham and Southampton. We principally aimed to determine through distribution methods the minimal clinically important difference (MCID) for the raw and centile MMN-RODS scores at different time-points during the studied period, in relation to the baseline score. We aimed to compare the sensitivity of the MCID cut-offs in our cohort, ascertained through raw scores, centile scores, as well as the available individualised cut-offs and to study MMN-RODS changes throughout the follow-up period and determine in retrospect, how MCID application may impact upon disease management.

## Materials and methods

We included consecutive subjects with a clinical diagnosis of MMN attending University Hospitals Birmingham UK, and University Hospital Southampton, UK between 2015 and 2025, meeting criteria for a diagnosis of definite, probable or possible MMN, as per European Federation of Neurological Societies/Peripheral Nerve Society (EFNS/PNS) 2010 Guidelines [8], and evaluated longitudinally through the MMN-RODS scale.

Treatment protocols used at initiation varied in between our 2 centres. Initiating immunoglobulin dose was with 2 doses of 2 g per kilogram of dosing weight or ideal body weight, at 2 centres. Through different methods, subsequent dose and frequency changes were decided on a case-by-case basis, depending on the maximal response attained and clinical stability, with, if felt to be required, incremental or decremental dosage. Decremental dosage was not performed in any subject at either centre, when the latest raw MMN-RODS was found improved, by any amplitude, compared to the previous evaluation, except when the maximal score had been attained.

Raw to centile MMN-RODS score conversion and individualised MCID (through MCID-SE ≥ 1.96) were kindly provided to us by the original study authors (Prof. Karin Faber, Maastricht University, Netherlands) [6]. We collected demographic data, disease phenotype, disease duration, baseline raw and centile MMN-RODS at first evaluation with the scale, latest recorded raw and centile MMN-RODS, interval between initial and latest assessment. Additional intermediate MMN-RODS raw and centile scores (up to 2 for each subject) and their timings were collected to assess for longitudinal score changes.

The MCID for the raw and centile MMN-RODS was determined through the ½ SD method. Proportions of subjects attaining MCID cut-off level for clinically-meaningful amelioration were ascertained for both raw and centile scales at the considered assessment timings, as well as through the single applicable individualised MCID applied at all assessments. We finally also proposed to ascertain eventual determinants of latest achieved functional status and total improvement amplitude from variables known in earlier disease stages, including age, gender, initial raw MMN-RODS, raw MMN-RODS at first intermediate assessment, raw MMN-RODS at second intermediate assessment, raw ∆MMN-RODS (defined as the change in MMN-RODS score) between initial and first intermediate assessment, and raw ∆MMN-RODS between the initial and second intermediate assessment.

Statistical analyses were performed with SPSS 28.0 (Armonk, USA). Comparison of proportions were performed by Fisher Exact tests and comparison of means by paired or independent T-tests, as applicable. Sensitivity analyses were performed through McNemar’s Tests. Associations were studied through Spearman’s Rank Correlation. Independent associations were sought through linear regression. Significance was set at *p* < 0.05, for all tests.

This analysis was conducted as part of registered and approved retrospective clinical audits of the diagnosis and management of MMN at University Hospitals Birmingham, UK and University Hospital Southampton, UK (Reg. no.: CARMS-20703 and SEV-0890, respectively). Audit does not require Ethics Committee approval in the UK.

## Results

### Baseline characteristics

We included 32 consecutive subjects with a diagnosis of MMN (16 subjects from Birmingham and 16 subjects from Southampton), meeting EFNS/PNS 2010 criteria. Female to male ratio was 1:1.29. Mean age was 60.0 years (SD: 11.6), mean age at onset was 47.7 years (SD: 11.0), and mean total disease duration was 149.8 months (SD: 80.4). Twenty-nine subjects (90.6%) had pure upper limb involvement and 3 (9.4%) had upper and lower limb involvement. As per EFNS/PNS 2010 criteria, 27 subjects (84.4%) had definite MMN, 1 (3.1%) had probable MMN, and 4 (12.5%) had possible MMN. Twenty subjects (62.5%) were already on treatment at initial assessment with the MMN-RODS, and 12 subjects (37.5%), were newly-diagnosed at the time of initial evaluation with the MMN-RODS.

### Progression of raw and centile MMN-RODS scores over the course of the study period

We considered initial, latest, as well as up to 2 intermediate scores available in our cohort, over the follow-up period. Data were available for one (31/32 subjects), or two (29/32 subjects) intermediate evaluations. The median time from initial assessment to the first intermediate evaluation was 17.0 months (31 subjects range: 3–49), from initial to the second intermediate evaluation of 54.1 months (29 subjects, range: 8–93), and from initial to the latest evaluation, of 74.6 months (32 subjects, range: 2–120).

**Fig. 1:**
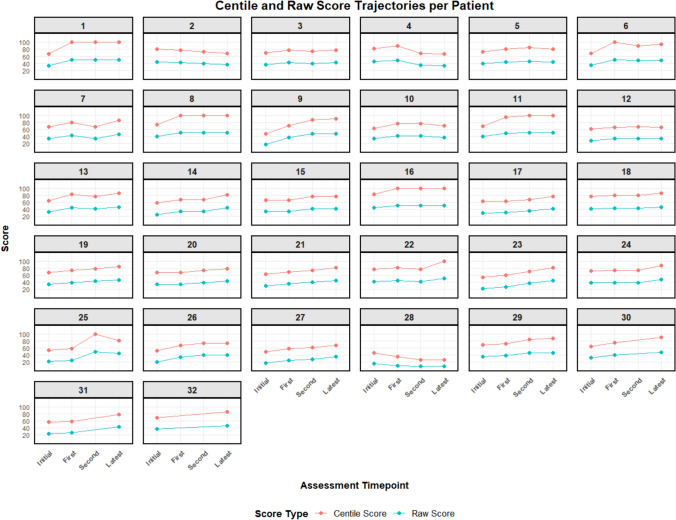
Centile and Raw MMN-RODS score trajectories per patient in 32 subjects with immunoglobulin-treated MMN, from Birmingham and Southampton, UK

Individual progression of raw and centile MMN-RODS scores in each studied subject over the study period, is shown in Fig. 1. At first intermediate assessment, improvement of any amplitude occurred in 27/31 (87.1%) subjects. At second intermediate assessment, improvement of any amplitude was noted in 24/29 (82.8%) subjects. At latest assessment, improvement of any amplitude was observed in 29/32 (90.6%) subjects.

**Fig. 2:**
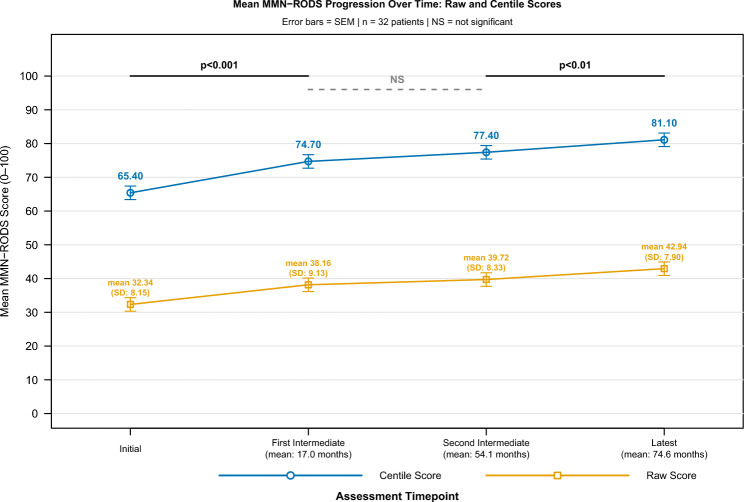
Mean raw and centile MMN-RODS score progression over time in 32 subjects with immunoglobulin-treated MMN, from Birmingham and Southampton, UK

Mean raw and centile MMN-RODS score evolution over the studied period, in the whole cohort is shown in Fig. 2. Mean MMN-RODS score progression over the study period is shown in Fig. 2. The raw MMN-RODS progressively improved from an initial mean of 32.34 (SD: 8.15), (i) to a mean of 38.16 (SD: 9.13) at first intermediate assessment, (ii) to a mean of 39.72 (SD: 8.33) at second intermediate assessment, and (iii) to a mean of 42.94 (SD: 7.90), at latest assessment. Hence, mean raw MMN-RODS improved significantly from initial to first intermediate assessment (*p* < 0.001), was comparable between first and second intermediate assessment (*p* = 0.21), then improved again significantly between second intermediate and latest assessment (*p* = 0.002). Similarly, the mean centile MMN-RODS improved at first intermediate evaluation compared to the initial assessment (74.7 [SD:14.7] vs. 65.4 [SD: 9.6]; *p* < 0.001), was comparable between first and second intermediate evaluations (74.7 [SD: 14.7] vs. 77.4 [SD: 15.1]; *p* = 0.28), then further improved between the second intermediate and the latest assessment (77.4 [SD: 15.1] vs. 81.1 [SD: 14.7]; *p* = 0.019).

### ∆MMN-RODS evolution and timing-specific MCID derivation through distribution methods at studied time-points

The mean raw ∆MMN-RODS was (1) from initial to first intermediate assessment of 5.97 (SD: 5.6), (2) from initial to second intermediate assessment of 7.38 (SD: 7.94), and (3) from initial to latest assessment, of 10.59 (SD: 8.76). Hence, the clinically-applicable distribution-based raw MCID (½SD) was 3, 4 and 5, at first intermediate, second intermediate and latest assessments, respectively.

The mean centile ∆MMN-RODS was (1) from initial to first intermediate assessment of 9.23 (SD: 10.41), (2) from initial to second intermediate assessment of 12.66 (SD: 13.77), and (3) from initial to latest assessment, of 16.03 (SD: 13.08). Hence, the clinically-applicable distribution-based centile MCID (½SD) was 6, 7 and 7, at first intermediate, second intermediate and latest assessments, respectively.

### Comparison of timing-specific raw MCIDs vs. timing-specific centile MCIDs vs. single applicable individualised MCIDs, through sensitivity analysis at studied time-points

**Fig. 3:**
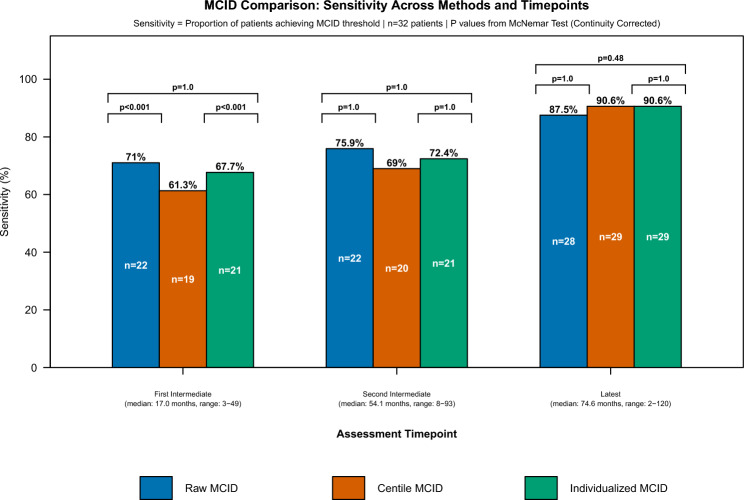
Comparison of MCID for the MMN-RODS: sensitivity through different methods and at different time points

The comparative sensitivity analysis at each studied assessment time, using the distribution-based, clinically-applicable raw and centile MCID for the timing under consideration, and the single applicable individualised MCID at all time-points, are summarized Table 1 and in Fig. 3.

**Table 1:** Application of raw, centile and individualized MCIDs at different assessment time-points during follow-up of 32 subjects with MMN from University Hospitals Birmingham and University Hospital Southampton, UK

	First Intermediate Assessment (median: 12.0 months)	Second Intermediate Assessment (median: 51.0 months)	Latest Assessment (median: 94.5 months)
Raw MMN-RODS MCID application (≥ 3, ≥ 4 or ≥ 5, for each evaluated time-point respectively)	22/31 (71.0%)	22/29 (75.9%)	28/32 (87.5%)
Centile MMN-RODS MCID application (≥ 6, ≥ 6 or ≥ 7, for each evaluated time-point respectively)	19/31 (61.3%)	20/29 ((69%)	29/32 (90.6%)
Individualised MMN-RODS MCID application	21/31 (67.7%)	21/29 (72.4%)	29/32 (90.6%)
MCID method for optimal sensitivity	Raw and individualised	All equivalent	All equivalent

At first intermediate assessment, the derived raw MCID ≥ 3 points offered a sensitivity of 22/31 (71.0%), the derived centile MCID of ≥ 6 points offered a sensitivity of 19/31 (61.3%) and the individualised MCID, a sensitivity of 21/31 (67.7%). At first assessment, the raw MCID and individualised MCID were both more sensitive than the centile MCID (McNemar’s Test [Continuity Corrected]: *p* < 0.001 and *p* < 0.001, respectively). Raw and individualised MCIDs were of equivalent sensitivity (McNemar’s Test [Continuity Corrected]: *p* = 1).

At second intermediate assessment, the derived raw MCID ≥ 4 points offered a sensitivity of 22/29 (75.9%), the derived centile MCID ≥ 7 points offered a sensitivity of 20/29 (69.0%), and the individualised MCID, a sensitivity of 21/29 (72.4%). At second intermediate assessment, raw, centile and individualised MCIDs all had equivalent sensitivity (McNemar’s Test [Continuity Corrected]; *p* = 1 for all comparisons).

At latest assessment, the derived raw MCID ≥ 5 points offered a sensitivity of 28/32 (87.5%), whereas both the derived centile MCID ≥ 7 points and the individualised MCID offered a sensitivity of 29/32 (90.63%). At latest assessment, raw, centile and individualised MCIDs all offered equivalent sensitivity (McNemar’s Test [Continuity Corrected]: *p* = 1 for raw vs. centile; *p* = 0.48 for raw vs. individualised; *p* = 1 for centile vs. individualised).

### Predictors of the total raw ∆MMN-RODS over the study period and of the latest achieved raw MMN-RODS score

From the available longitudinal outcome data, we in addition, attempted to ascertain eventual predictors of the latest achieved functional status, defined as the latest recorded MMN-RODS score, as well as of the overall amplitude of improvement over the study period, in our cohort. The findings of correlation studies and linear regression are summarized in Table 2.

**Table 2:** Significance results for Spearman’s Rank Correlations and for Independent Associations through Linear Regression, of total raw MMN-RODS improvement (Total raw ∆MMN-RODS) and of latest raw MMN-RODS score in 32 subjects with MMN from University Hospitals Birmingham and University Hospital Southampton, UK

	TEST	Initial raw MMN-RODS	Raw MMN-RODS at first intermediate assessment	Raw MMN-RODS at second intermediate assessment	Raw ∆MMN-RODS between initial and first intermediate assessment	Raw ∆MMN-RODS between initial and second intermediate assessment
Total raw ∆MMN-RODS	Spearman’s Rank Correlation	*p* = 0.027	*p* < 0.001	*p* < 0.001	*p* = 0.013	*p* < 0.001
	Linear Regression	NS	NS	*p* < *0.001*	NS	NS
Latest raw MMN-RODS score	Spearman’s Rank Correlation	*p* < 0.001	NS	NS	*p* = 0.002	*p* < 0.001
	Linear Regression	NS	NA	NA	NS	*p* < *0.001*

Italics and underlined values indicate the significant statistical results, of main importance as relating to linear regression

NS, non-significant; NA, non-applicable

The latest raw MMN-RODS score correlated with the initial raw MMN-RODS score (*p* = 0.027), with the raw MMN-RODS score at first intermediate assessment (*p* < 0.001), with the raw ∆MMN-RODS between initial and first intermediate assessment (*p* = 0.013), and the raw MMN-RODS score at the second intermediate assessment (*p* < 0.001). An independent association of the latest MMN-RODS score was found with the raw MMN-RODS score at second intermediate assessment, only (*p* < 0.001).

The total raw ∆MMN-RODS correlated with the initial raw MMN-RODS score (*p* < 0.001), the raw ∆MMN-RODS between initial and first intermediate assessment (*p* = 0.002) and the raw ∆MMN-RODS between the initial and second intermediate assessment (*p* < 0.001). An independent association of the total raw ∆MMN-RODS was found only with the raw ∆MMN-RODS between the initial and second intermediate assessment (*p* < 0.001).

Hence, neither the final achieved functional status, nor the total amplitude of improvement, were independently predicted by variables known in earlier disease stages, at initiation or at first intermediate assessment.

## Discussion

Although representing the only current disease-specific PROMS for MMN, the MMN-RODS scale has not been used widely in long-term studies, to date. Centile score transformation without MCID, and individualised MCIDs have been proposed by the authors having derived the scale, although have not been applied to date in clinical or research practice, to our knowledge [9]. As part of a study of long-term outcome and immunoglobulin dosing, we recently derived the centile MCID at a single, final, time-point, in this same cohort of subjects [7]. In that analysis, we observed progressive amelioration of mean MMN-RODS scores in this cohort. As here described in the current study, continuing amelioration occurred over a median follow-up of 74.6 months (6.2 years) with significant levels of improvement achieved both, early (within a year), as well as much later (in the last 4 years) during follow-up. These findings interestingly contradict previous reports of gradual decline despite continuing immunoglobulin treatment in MMN, additionally indicating that very delayed improvement is achievable. This may raise questions about the manner in which ascertainment of clinically meaningful improvement may most relevantly and optimally be considered at different illness stages, as previous studies, importantly, did not use the MMN-RODS. In addition, it is possible individualised immunoglobulin dosing regimens, adapted throughout the course of the disease as was the case for our patients, may have resulted in absence of functional decline. Future studies of immunoglobulin regimens tailored as per individual progression, with multiple outcomes including both patient-reported and strength measures, are needed to determine if this may impact upon MMN disease progression.

It is possible that our findings may in part, relate to the use of the MMN-RODS scale itself. As such, it is plausible that bias of patient ascertainment due to the nature of the PROM itself, as a result of desire to remain on immunoglobulin treatment or to be offered higher immunoglobulin doses, or of fear of immunoglobulin dose reduction, may all have contributed to our results. Hence, our results may raise concerns about the MMN-RODS, as adequate PROM in the longitudinal evaluation of subjects with treated MMN. It is in this regard however noteworthy, that although not performed concurrently to MMN-RODS recordings, Jamar grip strength also improved significantly in this cohort during this study period, as we have reported previously [7], supporting therefore the validity of the MMN-RODS score improvements observed.

This present study, in addition to the previous, sought to ascertain MCID variability at different assessment times, as well as compare raw vs. centile vs. individualised MCIDs at these different time-points. Our results mainly demonstrate the value of raw MMN-RODS scores and the variability of their MCIDs at different assessment time-points, which have important potential implications for clinical practice. A lower MCID cut-off, as may intuitively be expected in circumstances of continuing, progressive long-term improvement, was derived for earlier disease stages, whereas higher cut-offs were derived for later stages. As such, our results suggest that while an initial improvement ≥ 3 raw points may be meaningful in the first year of treatment, a total improvement ≥ 5 points is, on the other hand, may be required for confirmed clinical meaningfulness after 7–8 years of continuing therapy. Hence, whereas continuing treatment after just 3 raw points of amelioration appears justifiable early on, that may not be the case subsequently. In view of the prolonged treatment administered generally in MMN [10, 11], we believe this may be of help in long-term management decisions. We, furthermore, found that raw and individualised MCIDs were equivalent in sensitivity to detect meaningful change in earlier disease, and that both were better than the centile MCID. In later disease stages, the 3 methods had, on the other hand, equivalent sensitivity. This is clinically useful as suggests the appropriateness of direct application of collected raw scores at any assessment time during follow-up of subjects with MMN, thereby in practice, avoiding tedious and impractical centile transformation and ascertainment of subject-specific score changes, which do not appear to offer additional benefit.

We were unable to demonstrate the value of initial disability or of the amplitude of early treatment-induced changes, in independently predicting the total amelioration over the follow-up period or the final outcome. This finding is also of importance for immunoglobulin dosing decisions. The implication lies, for example, in a low MMN-RODS score pre-treatment, and/or an initially sub-optimal amplitude of improvement with immunoglobulin, not predicting a subsequently poor global level of improvement and/or poor final outcome. Striving for further benefit through continuing high dose treatment, dose escalation, and increased treatment frequency, may as a result, be more appropriate than early dose reduction and lengthening of treatment interval, as may frequently be considered instead, in the therapeutic management of such subjects.

Our study has several limitations, including its retrospective design and small sample size, the latter related to the low prevalence of MMN. Of note, the previous application of (individualised) MCIDs was performed on a smaller cohort of only 26 subjects [9]. As such, our findings may not be generalizable for these reasons. Also, we did not have systematically and concurrently administered additional scales such as the MRC sum score or grip strength. Data on patient global impression of change and quality of life measures had furthermore unfortunately, not been collected as part of routine care, and objective measurement of axonal loss progression through serial electrophysiology, was not conducted. Hence, associations and scale cross-validity could otherwise not be ascertained. With regards to the intrinsic characteristics of the PROM itself, multiple limitations to its application could not be considered in our analysis. As such, patient-related factors such as presence of co-morbidities, both physical and psychiatric [12, 13], variable perception of individual items on the scale, itself subject to many potential influences including social, cultural or geographic [14], health literacy and numeracy [15, 16], as well as anchoring bias (17), may all have impacted on the collected MMN-RODS scores.

Despite limitations, we believe that our results, derived from real-life routine clinical care data, are of interest in the application of the MCID concept in MMN, demonstrating the value of the raw MMN-RODS and its timing-specific MCIDs as equivalent in sensitivity, to the more difficult to apply, centile and individualised methods. The lack of value of initial scores and early improvement levels in predicting long-term prognosis may otherwise bring useful information to inform practical management modalities. Future prospective, collaborative multi-centre research studies of larger cohorts, using multiple patient-reported as well as strength measures, are desirable to further explore the optimal methods in monitoring clinically meaningful effects in MMN, as will be required both with immunoglobulin and novel therapies.

## Data Availability

The data that support the fi ndings of this study are available on request from the corresponding author. The data are not publicly available due to privacy or ethical restrictions.
